# Supplementary material for: Inhibition of tetrameric Patched1 by Sonic Hedgehog through an asymmetric paradigm
Source: Nat Commun. 2019 May 24;10:2320. doi: 10.1038/s41467-019-10234-9 (PMC6534611; doi:10.1038/s41467-019-10234-9)
Supplement: Supplementary file 1 — Supplementary Information [file 41467_2019_10234_MOESM1_ESM.pdf]

Supplementary Information

Inhibition of tetrameric Patched1 by Sonic Hedgehog through an asymmetric paradigm

Qian et al.

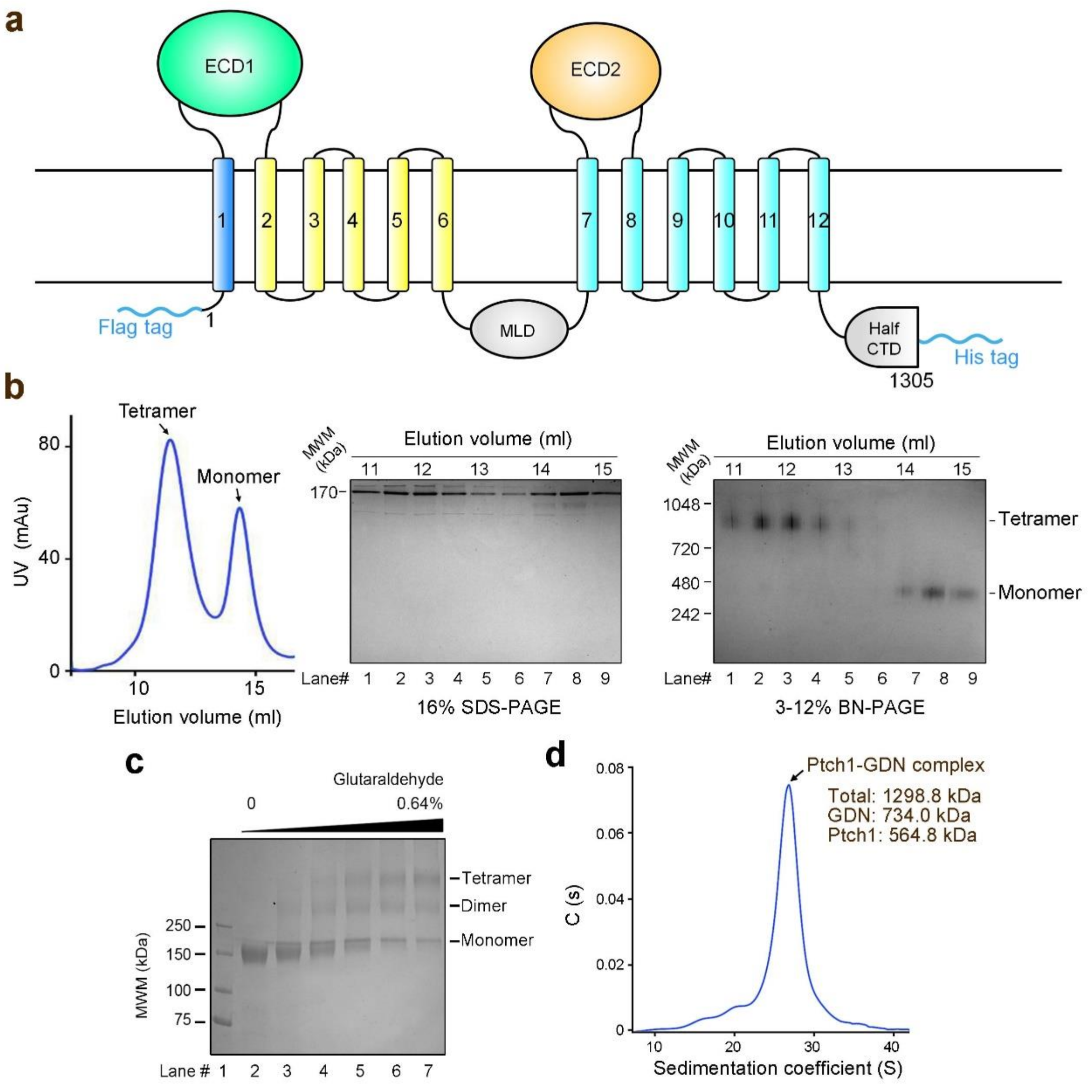

Supplementary Figure 1. Biochemical characterizations of the human Ptch1. a, Schematic illustration of the domain organization of Ptch1 and the construct used for recombinant protein expression. ECD: extracellular domain: MLD: middle-loop domain; CTD: C-terminal domain. **b**, Representative size exclusion chromatography (SEC) for the last step Ptch1 purification using GDN detergent. The peak fractions were subjected to SDS-PAGE or BN-PAGE. Molecular weight markers are indicated on the left side of the gels. Source data are provided as a Source Data file. **c**, Crosslinking of Ptch1 by glutaraldehyde. The increase in glutaraldehyde concentrations leads to the formation of crosslinked oligomers, which appears to be dimers and tetramers in SDS-PAGE. **d**, Sedimentation velocity analytical ultracentrifugation (AUC-SV) analysis of Ptch1/ShhN$_p$ complex. AUC-SV measured molecular weights support the tetrameric organization of Ptch1.

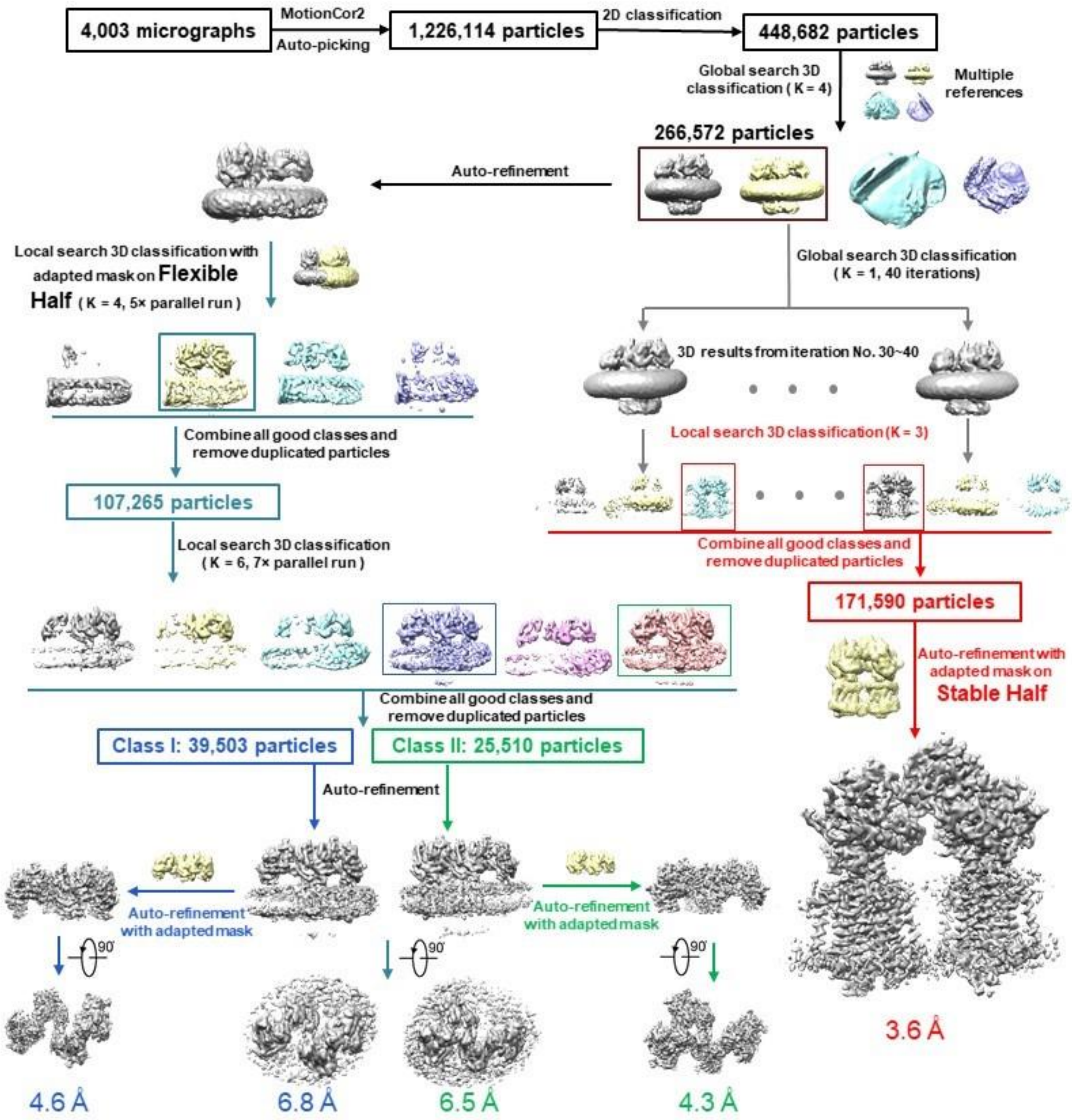

Supplementary Figure 2. Flowchart for cryo-EM data processing. Details are provided in Methods.

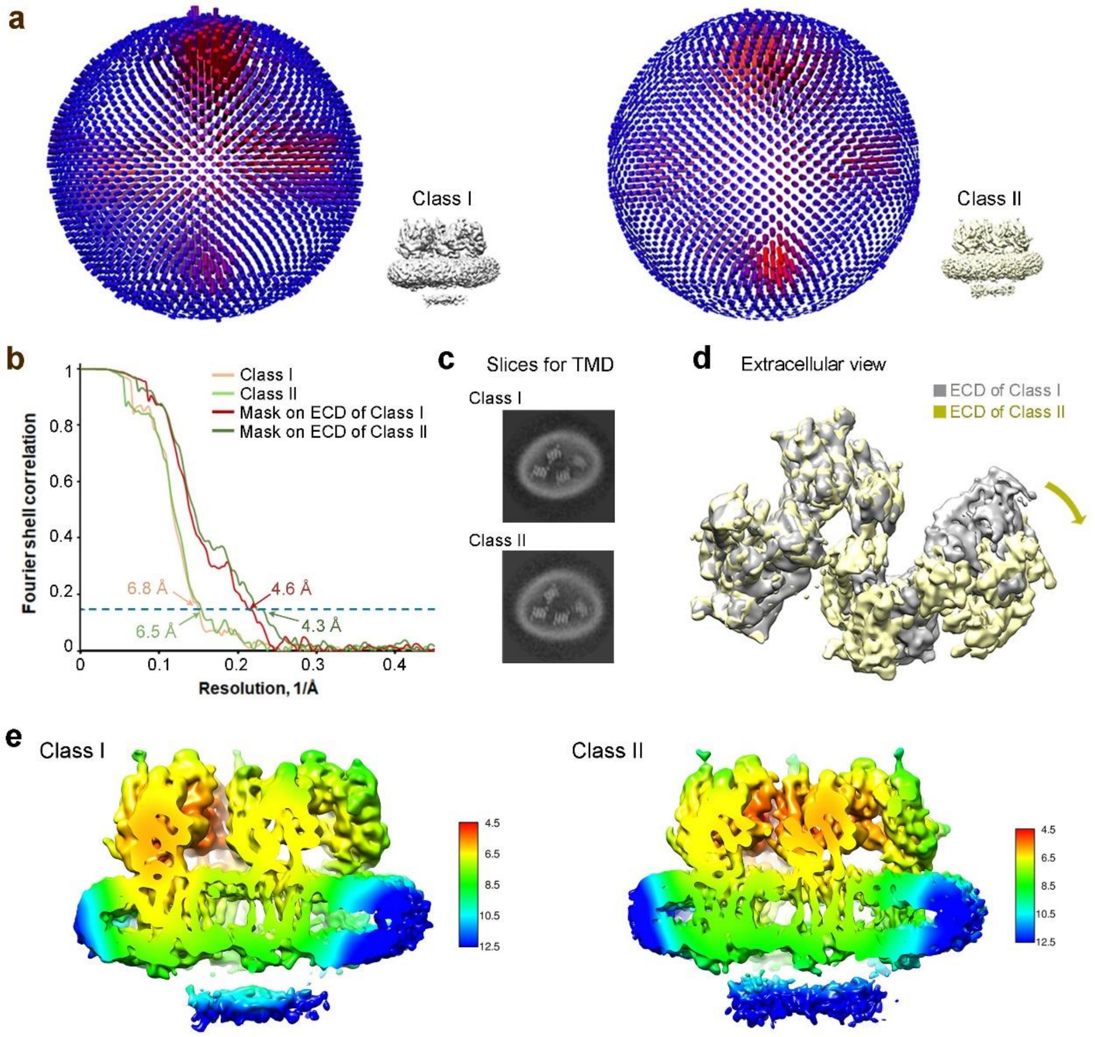

Supplementary Figure 3. Cryo-EM analysis of tetrameric Ptch1 in complex with ShhN$_{\mathbf{P}}$. **a,** Angular distributions for the two 3D reconstructions. **b,** The gold-standard Fourier shell correlation (FSC) curves for the density maps generated using Relion 2.0. **c,** Sliced sections of the transmembrane domain cut parallel to the membrane for the two reconstructions. **d,** Comparison of ECDs in the two reconstructions. Shown here is an extracellular view of the superimposed maps. **e,** The local resolution maps of the two classes calculated using Relion 2.0.

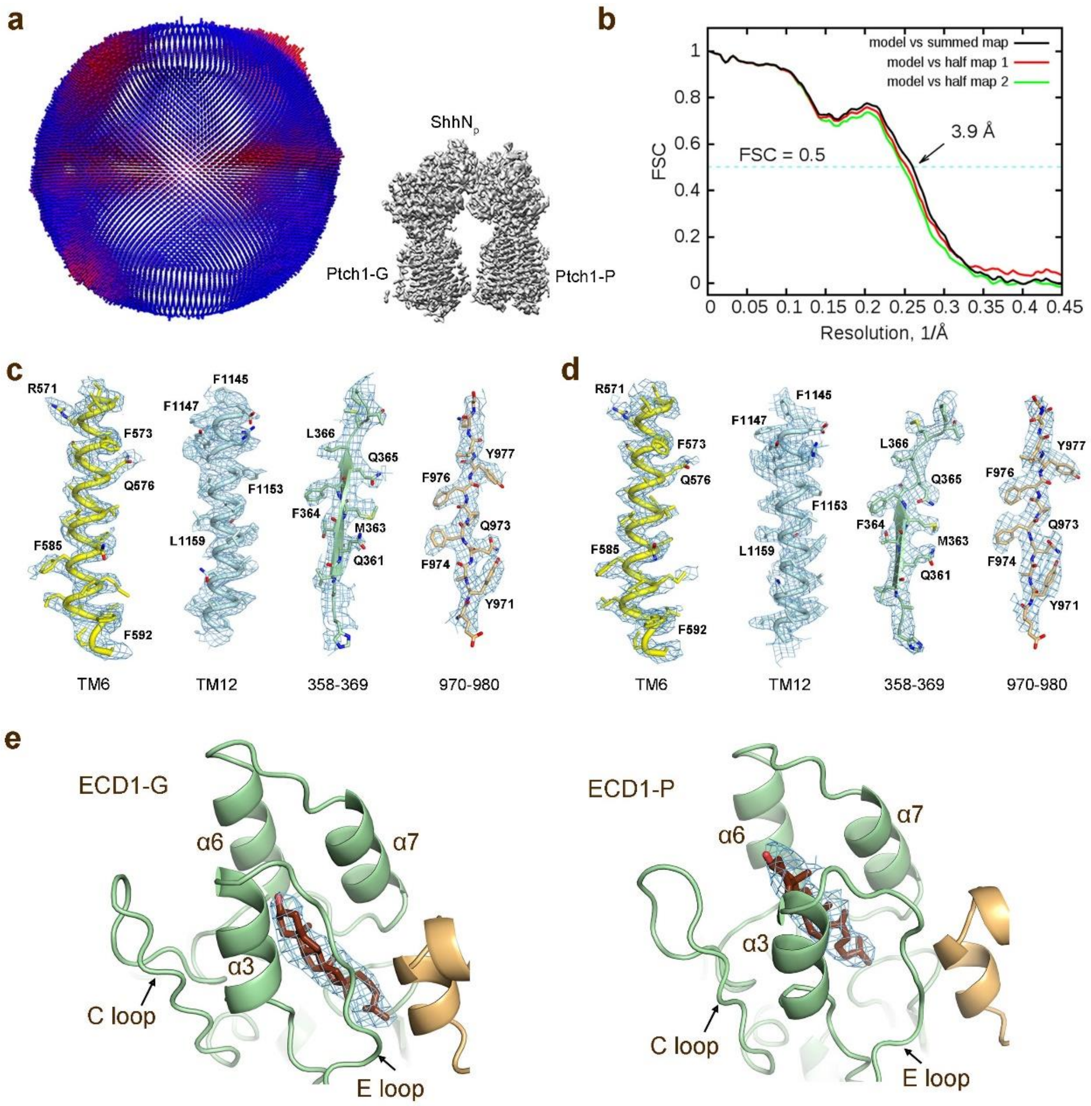

Supplementary Figure 4. Representative EM map for the 2:1 Ptch1-ShhN$_P$ complex. **a,** Angular distribution for the 3D reconstruction. **b,** The FSC curves calculated between the refined structure and the half map used for refinement (red), the other half map (green), and the full map (black). **c-d,** Structural models and corresponding densities for representative segments in Ptch1-G (**c**) and Ptch1-P (**d**). **e,** Two cholesterol-like densities embedded in ECD1 of Ptch1-G and Ptch1-P. The densities are shown as blue mesh at 6σ. A cholesterol is docked into each density.

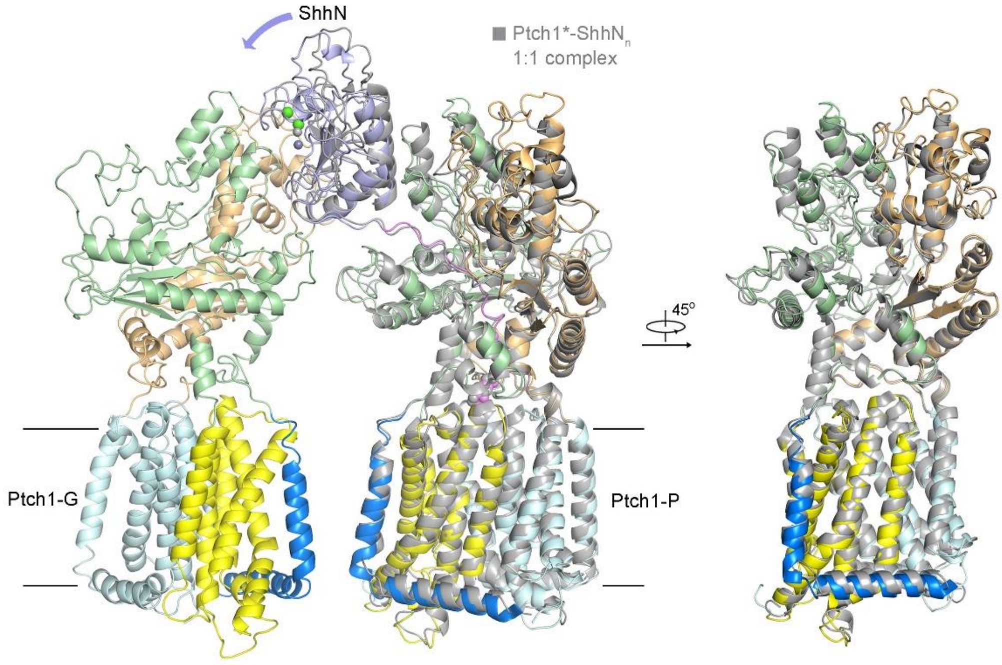

Supplementary Figure 5. Comparison of the structure of the 2:1 Ptch1-ShhN$_p$ complex with the 1:1 Ptch*-ShhN$_n$ complex. Superimposition of the 2:1 Ptch1-ShhN$_p$ and 1:1 Ptch1*-ShhN$_n$ complexes was made relative to Ptch1-P molecule. Ptch1-P in the 2:1 complex is nearly identical with Ptch1* in the 1:1 complex. ShhN$_p$ moves slightly away from Ptch1-P due to the interaction with Ptch1-G. The 2:1 Ptch1-ShhN$_p$ complex was domain colored, and the Ptch1*-ShhN$_n$ complex (PDB code 6D4J) was colored grey.

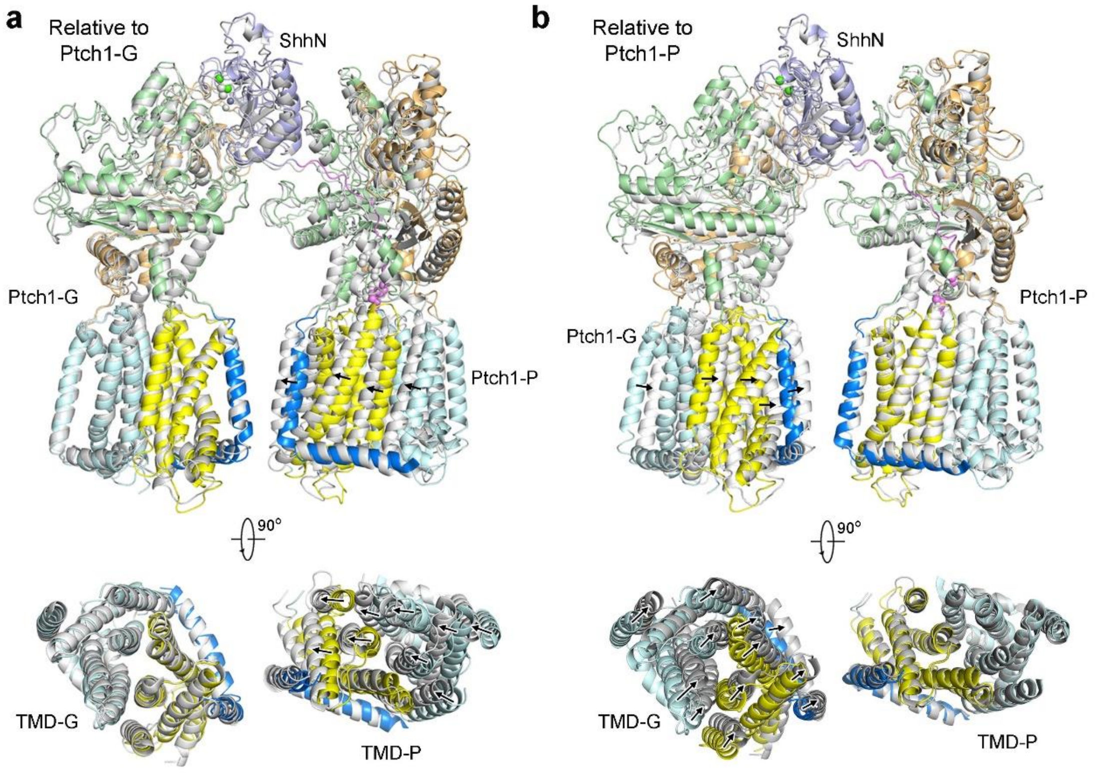

Supplementary Figure 6. Structural comparison of the 2:1 Ptch1-ShhN$_P$ and the recently published 2:1 Ptch1*-ShhN$_n$ complexes. The Ptch1-ShhN$_P$ complex was domain colored, and the Ptch1*-ShhN$_n$ complex was colored grey (PDB code 6E1H). The superimpositions were made relative to Ptch1-G (**a**) or Ptch1-P (**b**). Ptch1-G and Ptch1-P in our 2:1 complex are individually nearly identical with the two corresponding Ptch1* protomers, although the two Ptch1* protomers are closer to each other probably owing to the lack of restraint from the invisible intracellular domains.

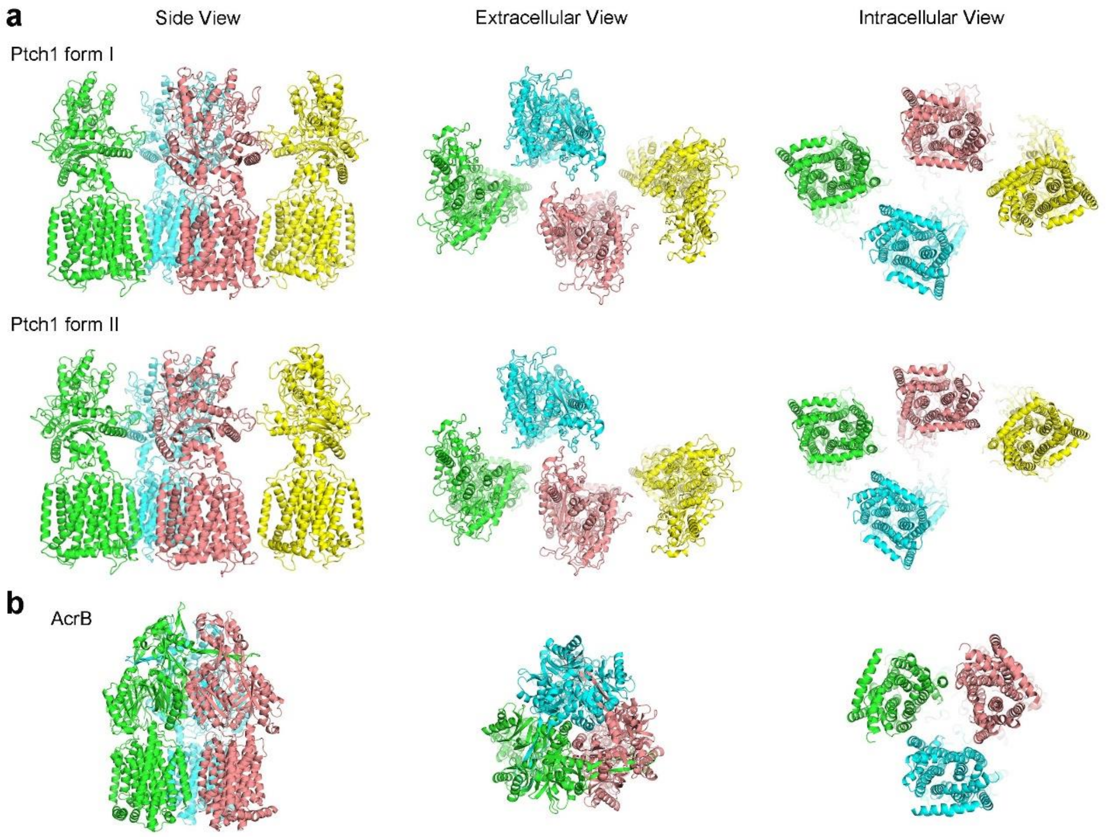

Supplementary Figure 7. The different oligomeric organizations of Ptch1 and AcrB. Shown here are the two tetrameric forms of Ptch1 (**a**) and the trimeric AcrB (PDB code 1IWG) (**b**). The side, extracellular and intracellular views are shown for each structure. Both the TMDs and ECDs of trimeric AcrB have extensive inter-protomer interactions to support the trimer formation. In contrast, the TMDs and ECDs of tetrameric Ptch1 barely contact each other.

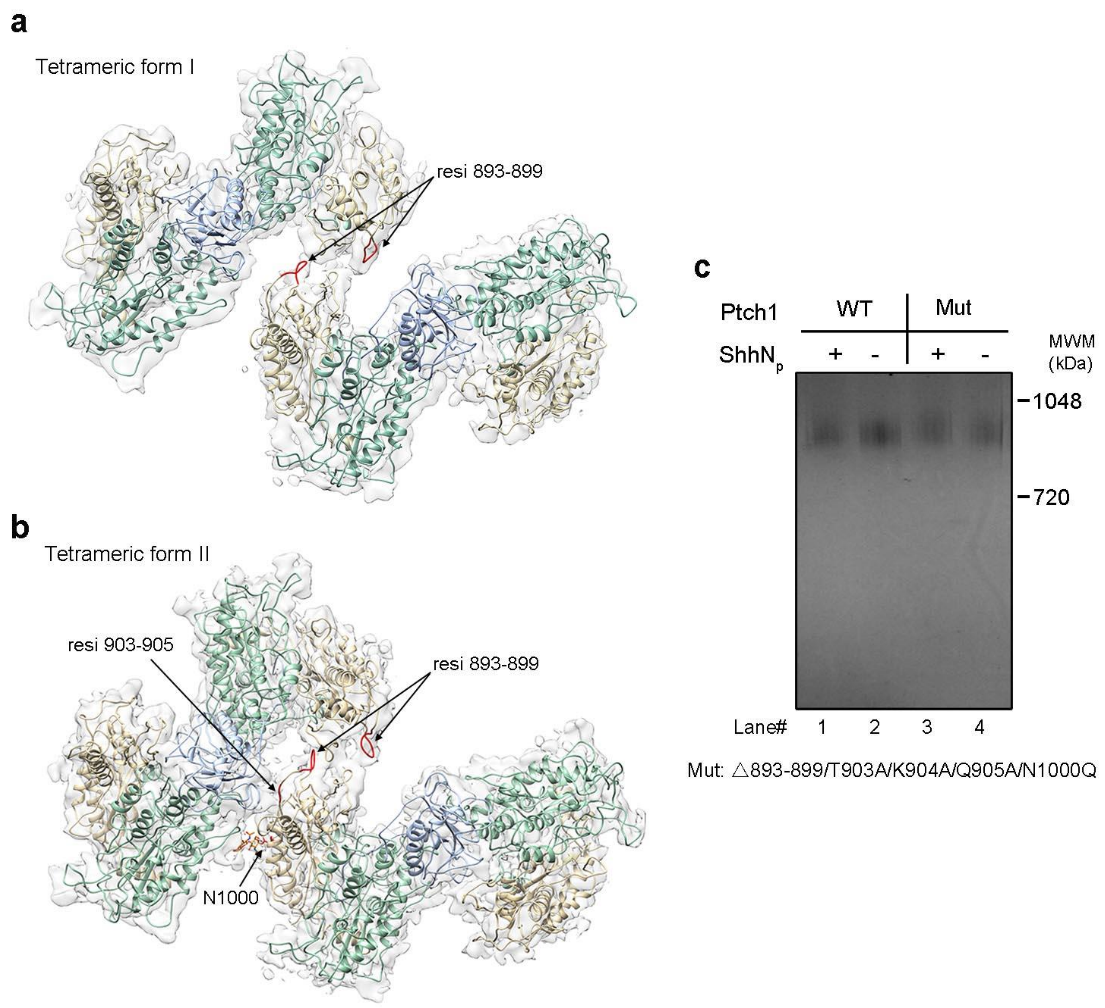

Supplementary Figure 8. The dimer-dimer interface of tetrameric Ptch1. **a**, The dimer-dimer interface in the tetrameric form I. **b**, The dimer-dimer interface in the tetrameric form II. **c,** A dimer-dimer interface variant failed to disrupt the tetrameric assembly of Ptch1 both alone and in complex with $ShhN_p$. Source data are provided as a Source Data file.

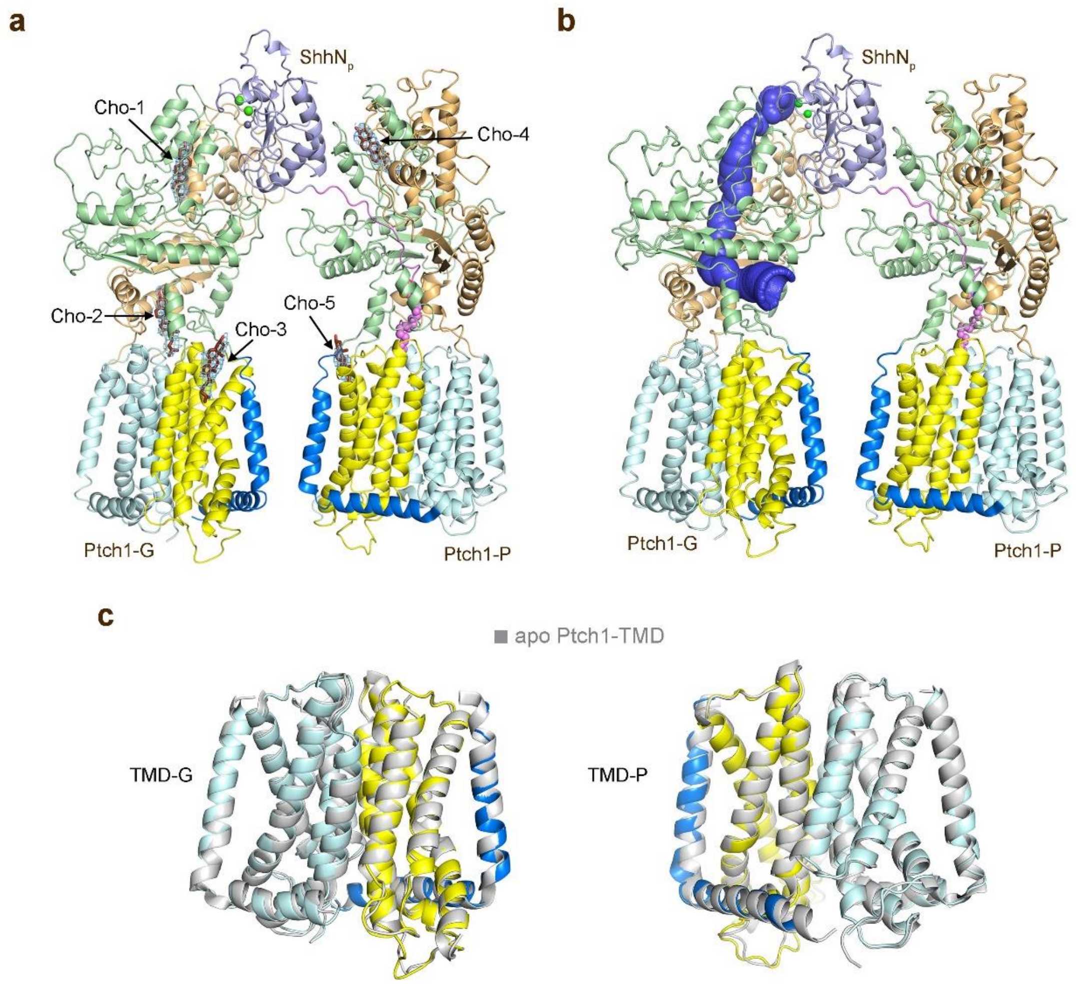

Supplementary Figure 9. Putative mechanism for the inhibition of Ptch1 by ShhN$_p$. **a,** There are five cholesterol-like densities in the 2:1 Ptch1-ShhN$_p$ structure. The densities, shown as blue mesh surrounding the docked cholesterol (labeled Cho-1 to Cho-5), are contoured at 6σ. **b,** A tunnel within the ECD of Ptch1-G. This tunnel, shown as dots resulted from calculation by HOLE, connects the cholesterol-like densities in ECD and SSD. **c,** Structural comparison of the TMDs of Ptch1-ShhN$_p$ (domain colored) and Ptch1 alone (grey, PDB code 6DMB). There are no substantial conformational changes between the TMDs of Ptch1 alone and the Ptch1-ShhN$_p$ complex.

	Ptch1-ShhN (2:1 complex)
**Data collection**	
EM	Titan Krios (Thermo Fisher)
Voltage (kV)	300
Detector	K2 Summit (Gatan)
Pixel size (Å/pixel)	1.114
Electron dose (e^-/Å^2)	50
Number of micrographs	4,003
**Reconstruction**	
Software	RELION 2.0
Number of used Particles	171,590
Symmetry	C1
Resolution	3.6 Å
Map sharpening B-factor (Å^2)	-156
**Model building**	
Software	Coot
**Refinement**	
Software	Phenix
**Model composition**	
Protein residues	2,153
Side chain	2,110
Sugar moieties	14
Ligands	5
Metal ions	3
**Validation**	
R.m.s deviations	
Bond length (Å)	0.02
Bond angle (º)	1.55
Ramachandran plot statistics (%)	
Preferred	86.98
Allowed	12.51
Outlier	0.51

Supplementary Table 1. Summary of data collection and model statistics.

Primer	Sequence (5'-3')
Ptch1-1-NotI-F	ATATAGCGGCCGCATGGCCTCTGCAGGCAATGC
Ptch1-1305-XhoI-R	TACGCCTCGAGGTCACGACGTGGTTGTTGTCCCTGAC
Ptch1-N1000Q-F	CACCATCTGCTCCCAATACACCAGCCTGGGTCTG
Ptch1-N1000Q-R	CAGACCCAGGCTGGTGTATTGGGAGCAGATGGTG
Ptch1-T903A/K904A/Q905A-F	CATCAGCCAGCTGGCCGCAGCTCGTCTGGTGGACGCTGAC
Ptch1-T903A/K904A/Q905A-R	GTCAGCGTCCACCAGACGAGCTGCGGCCAGCTGGCTGATG
Ptch1-△893-899(T903A/K904A/Q905A)-F	CTGCTGGTGCAGACCGGATCTAGCCAGCTGGCCGCAGCTCGTCTGG
Ptch1-△893-899(T903A/K904A/Q905A)-R	CCAGACGAGCTGCGGCCAGCTGGCTAGATCCGGTCTGCACCAGCAG
ShhN -1-NotI-F	AAATATGCGGCCGCATGTTGCTGCTGGCTCGT
ShhN -197-XhoI-R	GTACGCCTCGAGTCATCCACCGGACTTAGCAGC

Supplementary Table 2. Primers used in this study.
